# Efficient CRISPR-based genome editing for inducible degron systems to enable temporal control of protein function in large double-stranded DNA virus genomes

**DOI:** 10.71150/jm.2504008

**Published:** 2025-08-29

**Authors:** Kihye Shin, Eui Tae Kim

**Affiliations:** 1 Department of Microbiology and Immunology, Jeju National University College of Medicine, Jeju 63241, Republic of Korea; 2 Jeju Research Center for Natural Medicine, Jeju National University Core Research Institute, Jeju 63241, Republic of Korea

**Keywords:** CRISPR-Cas9 genome editing, human cytomegalovirus (HCMV), auxin-inducible degron (AID) system, integrase-deficient lentivirus (IDLV)

## Abstract

CRISPR-Cas9-based gene editing enables precise genetic modifications. However, its application to human cytomegalovirus (HCMV) remains challenging due to the large size of the viral genome and the essential roles of key regulatory genes. Here, we establish an optimized CRISPR-Cas9 system for precise labeling and functional analysis of HCMV immediate early (IE) genes. By integrating a multifunctional cassette encoding an auxin-inducible degron (AID), a self-cleaving peptide (P2A), and GFP into the viral genome via homology-directed repair (HDR), we achieved efficient knock-ins without reliance on bacterial artificial chromosome (BAC) cloning, a labor-intensive and time-consuming approach. We optimized delivery strategies, donor template designs, and component ratios to enhance HDR efficiency, significantly improving knock-in success rates. This system enables real-time fluorescent tracking and inducible protein degradation, allowing temporal control of essential viral proteins through auxin-mediated depletion. Our approach provides a powerful tool for dissecting the dynamic roles of viral proteins throughout the HCMV life cycle, facilitating a deeper understanding of viral pathogenesis and potential therapeutic targets.

## Introduction

Human cytomegalovirus (HCMV), a β-herpesvirus, is a double-stranded DNA virus with a large genome of approximately 236 kb, encoding at least 165 proteins, four non-coding RNAs, and 14 miRNAs (Forte et al., 2020; Van Damme and Van Loock, 2014). Of these genetic elements, approximately 30 are unique to β-herpesviruses, and many HCMV proteins play multiple roles during infection, complicating efforts to dissect their individual functions (Lin and Blissard, 2002; Rana and Biegalke, 2014).

HCMV exhibits a complex replication cycle, including lytic infection, latency, and reactivation, particularly in immunocompromised individuals. The regulation of this life cycle is tightly controlled by immediate early (IE), early, and late gene expression. Among these, IE genes play a pivotal role in initiating and regulating viral gene expression, making them central to pathogenesis and key targets for functional studies (Griffiths and Reeves, 2021).

Loss-of-function approaches, such as gene knockouts (KOs), have been widely used to study viral gene functions. However, for essential genes, KOs disrupt viral replication and hinder downstream analysis (Lin and Blissard, 2002). This significantly limits the ability to investigate proteins that are indispensable for viral fitness.

To overcome this limitation, we applied the auxin-inducible degron (AID) system, which enables conditional and reversible degradation of target proteins. This system utilizes the plant auxin pathway, in which the addition of auxin facilitates the interaction between AID-tagged proteins and the F-box protein OsTIR1, triggering proteasomal degradation via the SCF E3 ubiquitin ligase complex (Nishimura et al., 2009; Yesbolatova et al., 2020). By controlling the timing of auxin addition, this approach allows acute protein depletion at specific stages of infection, providing insights into dynamic protein function.

To facilitate the screening of recombinants, we used a self-cleaving P2A peptide to link AID with GFP. The P2A sequence ensures that GFP and the AID-tagged viral protein are expressed from a single transcript but cleaved into separate polypeptides (Liu et al., 2017). GFP serves as a visual marker for successful knock-ins, while the residual P2A tag does not interfere with protein degradation or function.

We first applied this AID-P2A-GFP system to tag the immediate early 1 (IE1) gene in HCMV. IE1 is a critical regulator of infection, modulating host immune responses, chromatin structure, and viral gene expression (Ahn et al., 1998; Reeves and Sinclair, 2013). Although its roles in antagonizing interferon signaling and PML nuclear bodies are well-characterized (Kim and Ahn, 2015), recent studies suggest that IE1 may contribute to viral persistence and reactivation (Adamson and Nevels, 2020; Collins-Mcmillen et al., 2019). However, its essential nature precludes the use of traditional knockout approaches.

Traditionally, bacterial artificial chromosome (BAC)-based recombineering has been used for genetic manipulation of HCMV (Warden et al., 2011), but this method is time-consuming, labor-intensive, and often unsuitable for manipulating clinical strains or performing high-throughput studies. CRISPR-Cas9 technology has emerged as a powerful alternative, offering precise genome editing capabilities. However, applying CRISPR-Cas9 to large DNA viruses like HCMV poses challenges, particularly in achieving efficient homology-directed repair (HDR) and maintaining cell viability.

Various approaches have been developed to achieve knock-in modifications in viral genomes via CRISPR-Cas9-induced HDR (Leal et al., 2024; Liao et al., 2024). These approaches differ in the forms in which CRISPR components are delivered. For example, Cas9 can be introduced as DNA for intracellular expression or as a purified protein, and single-guide RNA (sgRNA) can be delivered either as DNA or in vitro-transcribed RNA. The HDR donor template may take the form of single-stranded DNA (ssODNs), single-stranded RNA (ssRNA), linear double-stranded DNA (dsDNA), or plasmid DNA, each with specific trade-offs. The choice of format depends on experimental objectives and available resources (Liao et al., 2024).

While these advancements offer promising avenues, the development of efficient and adaptable genome editing tools for the conditional analysis of essential genes in large DNA viruses remains limited. In this study, we introduce a CRISPR-Cas9-based knock-in platform for HCMV that incorporates an auxin-inducible degron system for temporal control of protein stability. We demonstrate its utility by targeting the essential IE1 and IE2 genes, achieving conditional tagging without the use of complementing cell lines, thereby providing a broadly applicable strategy for dissecting gene function across the HCMV life cycle.

## Materials and Methods

### Cell culture and virus

Human foreskin fibroblast HFF-1 cells (ATCC, SCRC-1041) were used for HCMV infection and cultured in a 5% CO_2_ incubator at 37°C. Cells were grown in Dulbecco’s modified Eagle’s medium (DMEM; Welgene, LM001-05) supplemented with 10% fetal bovine serum (FBS; Invivogen, F0900-050) and 100 U/ml penicillin plus 100 µg/ml streptomycin (Gibco, 15140-122). HCMV strain Toledo is used for all experiments and propagated using HFFs.

### Construction of CRISPR-Cas9 and HDR donor template plasmid

To construct the CRISPR-Cas9 plasmid system, we used an all-in-one lentiviral vector co-expressing Cas9 and single-guide RNA (sgRNA) (addgene #48138). The sgRNA sequence was inserted into the vector by *BsmBI* restriction enzyme (Enzynomics, R057H) digestion and ligation of annealed primers.

The HDR donor template was designed to contain homologous arm sequence 1 (HAS1), the AID-P2A-GFP cassette, and homologous arm sequence 2 (HAS2). HAS1 and HAS2 were amplified from HCMV genomic DNA. The AID-P2A-GFP sequence was synthesized by TWIST Bioscience. mAID sequence is used for AID coding gene for AID-P2A fusion and is listed in Table 1. The pLenti6.3/V5-DEST vector (Invitrogen, V53306) was used as the backbone and amplified by PCR to retain the long terminal repeat (LTR) regions. The final HDR donor template was assembled using the Gibson Assembly method with the NEBuilder® HiFi DNA Assembly Master Mix (New England Biolabs, E2621L) by combining HAS1, AID-P2A-GFP, HAS2, and the pLenti6.3 backbone.

### Lentivirus production and transduction

Lentiviral particles were produced by transfecting HEK293FT (Invitrogen, R70007) cells with packaging plasmids. Cells were seeded in 6-well plates and cultured to approximately 70% confluency in DMEM (Welgene, LM001-05) supplemented with 10% Cosmic Calf Serum (HyClone, SH30087.03) at 37°C in a humidified incubator with 5% CO₂. The transfection mixture consisted of 1 µg of transfer vector, 0.75 µg of pCMVΔR8.74 or pCMVΔR8.74 D64V, and 0.25 µg of pMD.G2. At 48 h post-transfection, the culture supernatant was harvested and filtered through a 0.45 µm syringe filter (Jet Bio-Filtration, J1.F404.013N) to remove cellular debris. The filtered viral supernatants were then supplemented with polybrene at a final concentration of 10 µg/ml to enhance transduction efficiency and applied to target cells. The lentiviral titers were determined by quantitative PCR (qPCR) targeting the WPRE sequence. Genomic DNA (gDNA) was extracted from cells transduced with the lentivirus, and the copy number of viral cDNA was quantified by WPRE-specific qPCR. A plasmid containing the WPRE sequence was used as a standard for quantification. The resulting titers ranged from 5 × 10^5^ to 1 × 10^7^ copies/ml, and lentiviral preparations within this range were used in this study.

### Mutagenesis of D64V integrase mutation

The D64V point mutation was introduced into the pCMVΔR8.74 packaging plasmid via site-directed mutagenesis. Mutagenic primers were designed to substitute the wild-type integrase sequence with the D64V variant. PCR amplification was carried out using these primers, and resulting plasmid was validated by Sanger sequencing to confirm the presence of the desired mutation.

### Optimization of knock-in efficiency

To optimize knock-in efficiency, lentiviral transduction was performed in 12-well plates at MOIs of 1, 3, 6, and 12. In parallel, the ratio of donor template lentivirus to Cas9-sgRNA lentivirus was systematically adjusted. GFP-positive plaques were visualized and counted using an EVOS M7000 imaging system (Invitrogen), and GFP-positive cells were quantified by flow cytometry using a FACS analyzer (CytoFLEX, Beckman Coulter).

### Generation of TIR1-expressing cells

The plasmid pAAV-hSyn-OsTIR1(F74G) was obtained from Addgene (plasmid #140730) (Yesbolatova et al., 2020). The OsTIR1(F74G) coding sequence was amplified by PCR and subcloned into the pDONR221 entry vector (Invitrogen, #12536017). Gateway LR recombination was then performed to transfer the insert into the destination vector pLenti6.3/V5-DEST. The resulting construct was used to transduce human foreskin fibroblast HFF-1 cells (ATCC, SCRC-1041). Successfully transduced cells were selected with 2 μg/ml of blasticidin (Sigma-Aldrich, SBR00022) for downstream applications.

### Plaque purification

GFP-positive plaques were isolated under a fluorescence microscope using 0.3% agarose in DMEM overlay. Each plaque was picked and subjected to six rounds of plaque purification to ensure clonal isolation. Purified viruses were expanded in HFFs and validated by PCR, sequencing, and western blot for downstream analyses.

### Validation of knock-in and AID system

Genomic DNA was extracted from cells, and the target region was amplified using Phusion^TM^ Plus DNA polymerase (Thermo Scientific, F630S). The PCR product was cloned into a T-vector and subjected to Sanger sequencing to confirm successful knock-in. To validate expression of the AID-P2A-GFP construct, western blot analysis was performed using the IE1-specific antibody, which was kindly provided by Professor Jin-Hyun Ahn (Sungkyunkwan University) (Shin et al., 2012), with GAPDH as a loading control.

### Statistical analysis

All data were analyzed using GraphPad Prism software (v8.0.1). Quantitative results are presented as the mean ± standard deviation (SD). Statistical significance was assessed using either Student’s *t*-test or one-way ANOVA, as appropriate. *p*-value of < 0.05 was considered statistically significant.

## Results

### Construction and application of lentiviral vectors for genome editing

**Fig. 1.**
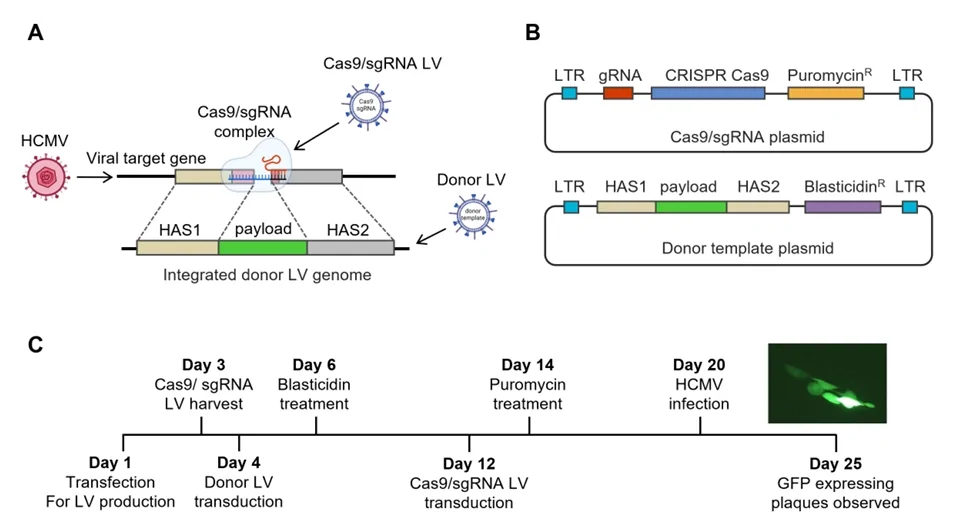
CRISPR-Cas9 knock-in strategy for HCMV genome editing using lentiviral delivery system. (A) Schematic of lentivirus-mediated delivery of CRISPR-Cas9 for precise HCMV genome editing. Cas9 and sgRNA are delivered via one lentiviral vector (Cas9/sgRNA LV), while the donor template carrying the knock-in cassette flanked by homologous arms (HAS1 and HAS2) is delivered via a separate donor lentivirus (Donor LV). Upon co-transduction, the Cas9-sgRNA complex induces a double-strand break at the viral target gene, facilitating homology-directed repair (HDR) using the integrated donor template. (B) Vector map of lentiviral transfer constructs. The pLentiCRISPR vector expresses both sgRNA and Cas9 along with a puromycin resistance marker. The donor template vector (pLenti6.3) includes HAS1, payload, HAS2, and a blasticidin resistance cassette to enable HDR-based knock-in into the HCMV genome. (C) Timeline of the experimental workflow. HEK293FT cells are transfected on Day 1 to produce lentiviruses. Lentiviral vectors are used for donor transduction (Day 4) and Cas9/sgRNA transduction (Day 12) in human fibroblasts. Blasticidin and puromycin selection are applied on Days 6 and 14, respectively. HCMV infection occurs on Day 20, with GFP-positive plaques observed by Day 25.

To deliver the Cas9 protein, sgRNA, and the HDR donor template into cells, we employed a lentiviral delivery system. Lentiviruses enable stable expression of CRISPR components by integrating their genomes into the host DNA. This integration ensures continuous expression of both Cas9 and sgRNA, as well as a sustained presence of the HDR donor template in host cells to facilitate the DNA repair process (Fig. 1A). Because many HCMV-susceptible cell types are difficult to transfect, lentiviral vectors provide a more efficient delivery method owing to their high transduction efficiency.

We first generated two lentiviral transfer vectors: one encoding Cas9 and sgRNA, and the other carrying the HDR donor template. The donor template included homologous arm sequences (HAS1 and HAS2) flanking the target site, as well as the payload sequence for knock-in. Although previous studies have suggested that optimal HAS lengths range from 500 to 1,000 bp (Wang et al., 2017), our preliminary results showed no significant difference in efficiency between 500 and 1,000 bp arms. Nevertheless, we selected 1,000 bp arms for all subsequent experiments. These vectors were co-transfected with packaging and envelope plasmids into HEK293FT cells to produce the lentiviral particles (Fig. 1B).

To initiate CRISPR-Cas9-mediated genome editing, HFFs were transduced with the Cas9-sgRNA lentivirus and selected with blasticidin to enrich successfully transduced cells. These cells were then infected with the HDR donor template lentivirus, followed by puromycin selection to generate stable cell lines expressing Cas9 and sgRNA while harboring the integrated HDR donor template. The resulting stable cells were then infected with HCMV Toledo strain at an MOI of 3 (Fig. 1C).

Although GFP-expressing plaques were successfully obtained using this lentiviral CRISPR-Cas9 HDR system, the efficiency was extremely low, with only one to two GFP-positive plaques per well in a 6-well plate (data not shown). Moreover, the stable cell lines generated by lentiviral integration exhibited noticeably slow growth, which significantly delayed the overall experimental timeline. These results highlight the inefficiency and labor-intensive nature of this method, underscoring the need for a more optimized strategy.

### Enhancing efficiency with integrase-deficient lentivirus (IDLV) systems

To improve editing efficiency and accelerate the workflow, we employed integrase-deficient lentivirus (IDLV) vectors. IDLVs retain the structural components of conventional lentiviruses but carry a D64V mutation in the integrase gene, which prevents them from integrating into the host genome (Gaur and Leavitt, 1998). After transduction, the IDLV genome is reverse-transcribed into double-stranded DNA, which is transiently expressed. This DNA allows for Cas9 protein production and sgRNA transcription, enabling CRISPR-mediated genome editing without permanent genomic integration. The HDR donor template is also delivered as part of the IDLV RNA genome and, once reverse-transcribed, functions as the repair template for HDR.

**Fig. 2.**
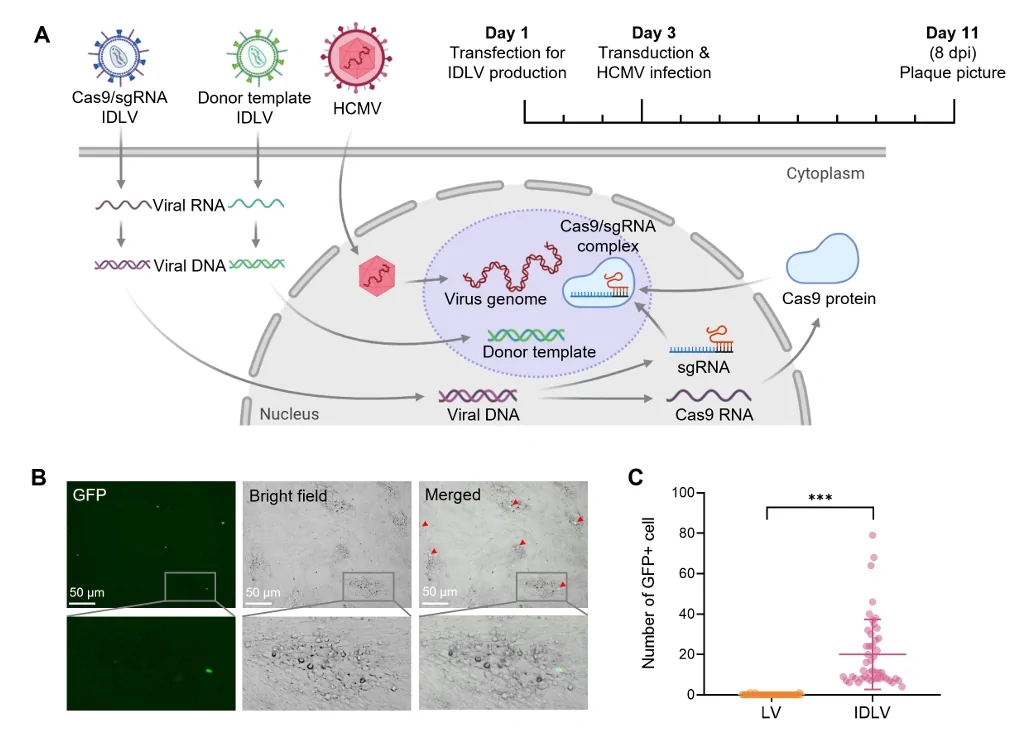
Enhanced genome editing efficiency in HCMV using IDLV for CRISPR-Cas9-mediated knock-in. (A) Schematic representation of the IDLV-based CRISPR knock-in strategy. Cas9/sgRNA and HDR donor templates are delivered via IDLVs, enabling transient expression without genomic integration. Upon co-delivery with HCMV, the Cas9-sgRNA complex induces a double-strand break at the target site in the viral genome, and the donor template facilitates HDR. (B) Representative fluorescence microscopy images showing successful knock-in of GFP at the C-terminus of the IE gene in HCMV. GFP-positive cells (green) indicate edited viruses. Red arrowheads mark GFP-expressing plaques, illustrating the enhanced editing efficiency of the IDLV approach, even in the presence of unedited cells. (C) Quantification of GFP-positive cells at 7 days post HCMV infection comparing conventional integrating lentivirus (LV) and IDLV. A significant increase in knock-in efficiency is observed with IDLV delivery. Data represent individual counts from n = 40–50 fields; bars indicate mean ± SD. ^***^p < 0.001 by one-way ANOVA.

To initiate HDR using IDLVs, we generated both Cas9-sgRNA and HDR donor template vectors using a packaging plasmid containing the D64V integrase mutation. HFFs were co-infected with these IDLVs along with the HCMV Toledo strain at an MOI of 3 (Fig. 2A). Interestingly, the number of resulting plaques indicated that the effective MOI was significantly lower than expected (data not shown). We later determined that this reduction was due to polybrene, which was added to facilitate lentiviral transduction but unintentionally inhibited HCMV infection. Nevertheless, GFP-positive plaques were observed as early as six days post-infection, and the editing efficiency was significantly improved compared to that of the conventional integrating lentiviral vector method. IDLV-mediated editing resulted in a much higher frequency of GFP-positive cells (Fig. 2B), indicating enhanced HDR efficiency. Furthermore, quantitative analysis confirmed this improvement, with a significantly increased number of GFP-positive cells counted at 7 days post-infection (n = 40–50) (Fig. 2C). In contrast to the integrating lentiviral vector approach, which yielded only one or two GFP-positive plaques out of 100, nearly all plaques from the IDLV system exhibited GFP expression.

The IDLV approach offers several advantages over conventional integrating lentivirus-based genome editing. Because it avoids integration into the host genome, it better preserves cell viability and reduces the risk of off-target effects associated with permanent vector insertion. In addition, the workflow is considered faster, as it bypasses the need to establish stable cell lines, significantly shortening the overall genome editing timeline (Fig. 2A).

Despite these improvements, HDR efficiency remained below ideal levels. To understand the limiting factors, we investigated the form and intracellular localization of the HDR donor template. Previous studies have shown that HDR efficiency depends heavily on the format of the donor template: single-stranded DNA (ssDNA) typically yields the highest efficiency, followed by linear double-stranded DNA (dsDNA), while plasmid DNA is usually the least effective. These insights led us to refine the design of our donor template to enhance its performance in HDR-driven genome editing.

### Incorporation of CRISPR target “cut site” in HDR donor templates

In our experimental system, the donor template was incorporated into the lentiviral RNA genome. Following reverse transcription, it was converted into a large double-stranded DNA molecule containing viral long terminal repeats (LTRs) and other cis-acting elements. This structural complexity likely contributed to the limited HDR efficiency observed.

**Fig. 3.**
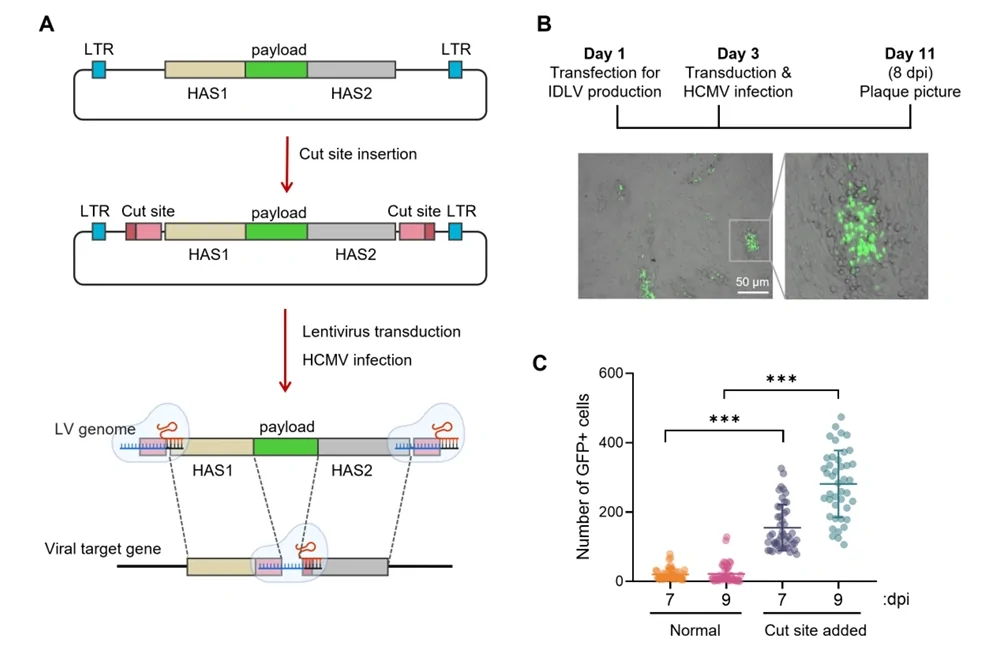
The inclusion of CRISPR cut sites in HDR donor templates enhances knock-in efficiency. (A) Schematic illustration of the HDR donor template strategy. The top panel shows the original pLenti6.3-based donor template lacking CRISPR cut sites. The middle panel includes donor templates with CRISPR target sites flanking both ends (HAS1 and HAS2), enabling simultaneous cleavage of both the donor and viral target sequences. The bottom panel illustrates donor integration into the viral genome through homology-directed repair (HDR) following lentiviral delivery and reverse transcription. (B) (Top) Experimental timeline for IDLV-based genome editing in HCMV. (Bottom) Fluorescence microscopy images show increased numbers of GFP-positive plaques in the donor template group containing cut sites, indicating improved editing efficiency. The right panel displays a magnified view of a representative plaque exhibiting strong GFP expression. (C) Quantitative analysis of knock-in efficiency. GFP-positive cells were counted at 7 and 9 dpi under normal and cut-site-included donor template conditions. Data are presented as individual values from n = 40–50 fields per condition. ^***^p < 0.001 by one-way ANOVA.

To address this, we redesigned the donor template by adding CRISPR target “cut sites” flanking both ends of HAS1 and HAS2. These cut sites matched the Cas9 target site located at the C-terminal region of the IE1 gene, allowing the Cas9-sgRNA complex to cleave both the HCMV genome and the donor template at homologous arms (Fig. 3A). This dual cleavage not only linearized the donor template at the CRISPR target site where viral replication occurs, enhancing local concentration and spatial targeting.

To validate this approach, we incorporated the modified donor template into the IDLV vector and co-infected HFFs with this construct along with Cas9-sgRNA-expressing IDLVs and HCMV (Fig. 3B). The inclusion of cut sites led to a dramatic increase in GFP-positive cells, confirming improved HDR efficiency. Without cut sites, only one to two GFP-positive cells were observed per plaque, whereas inclusion of the cut sites resulted in markedly higher numbers of GFP-positive cells within each plaque (Fig. 3B and 3C).

These results underscore the importance of donor template design in maximizing the efficiency of HDR-based genome editing and highlight the utility of CRISPR-targeted linearization to enhance delivery precision and effectiveness.

### Enhancing HDR efficiency with optimized conditions

To further improve HDR efficiency under more practical and scalable conditions, we systematically evaluated key parameters affecting recombination. These included the ratio between Cas9-sgRNA IDLV and donor template IDLV, as well as the MOI of HCMV.

**Fig. 4.**
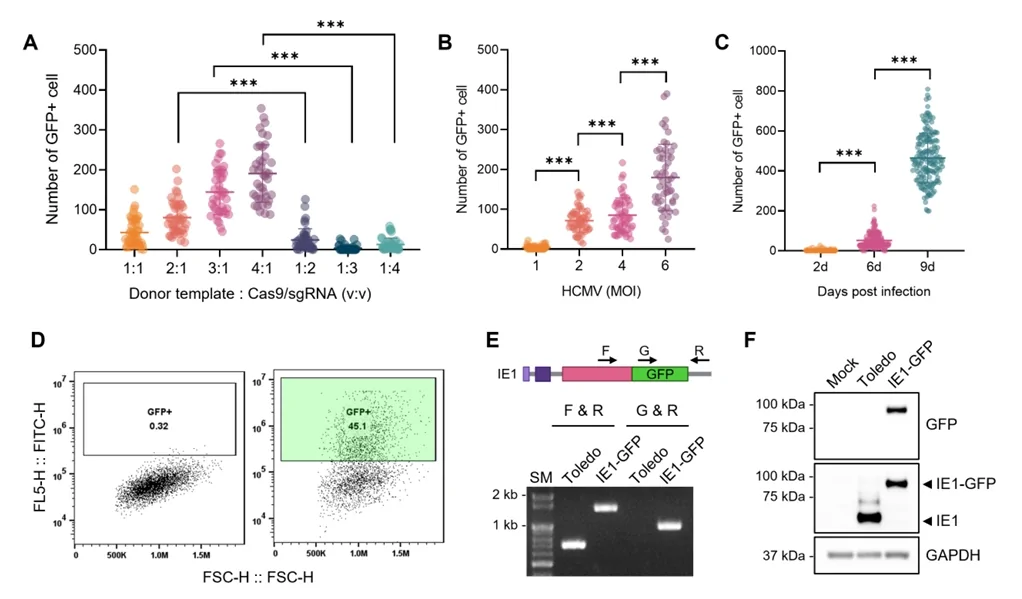
Optimization of CRISPR-Cas9 genome editing conditions in HCMV using IDLV. (A) Effect of varying donor template IDLV to Cas9/sgRNA IDLV ratios on HDR efficiency. GFP-positive cells were quantified at 7 dpi to assess knock-in rates. (B) Evaluation of HDR efficiency across different multiplicities of infection (MOI) for HCMV. Cells were infected at MOIs of 1, 2, 4, and 6, and GFP-positive cells were counted at 7 dpi. (C) Time-course analysis of GFP-positive cells at 2, 6, and 9 dpi. The increasing numbers indicate viral replication as well as progressive genome editing over time. (D) Flow cytometry analysis of GFP-positive cells before (left) and after (right) genome editing. The edited population displays a marked increase in GFP expression (~45%), confirming successful knock-in. (E) PCR validation of IE1-GFP knock-in using two primer sets (F & R and G & R) to distinguish wild-type and knock-in alleles. PCR products from IE1-GFP-infected cells confirm accurate integration at the IE1 locus. (F) Western blot analysis showing expression of the IE1-GFP fusion protein. GFP and IE1 were detected in cells infected with the genome-edited virus, whereas only untagged IE1 was detected in wild-type HCMV (Toledo)-infected cells. GAPDH was used as a loading control. ^***^p < 0.001 by one-way ANOVA.

We first examined the impact of varying the ratio of donor template IDLV to Cas9-sgRNA IDLV. HFF cells were co-infected with lentiviruses at different ratios (1:1, 1:2, 1:3, 1:4, 2:1, 3:1, and 4:1). GFP-positive cells were quantified at 7 and 9 days post-infection (dpi) to assess HDR efficiency. The results revealed a positive correlation between donor template abundance and recombination efficiency, with the 4:1 ratio yielding the highest number of GFP-positive cells (Fig. 4A). In contrast, increasing the Cas9-sgRNA IDLV beyond a certain threshold did not further enhance editing efficiency, indicating that donor template availability was the limiting factor (Fig. 4A).

We next assessed whether the MOI of HCMV influences recombination outcomes. HFF cells were infected with HCMV at varying MOIs (1, 2, 4, and 6) in combination with IDLV co-transfection. A clear MOI-dependent increase in GFP-positive cells was observed (Fig. 4B), suggesting that higher viral loads promote greater recombination, possibly by expanding the number of viral replication compartments accessible for CRISPR activity.

Time-course analysis further supported this trend, revealing a progressive increase in GFP-positive cells at 2, 6, and 9 days post-infection, consistent with viral propagation and accumulation of editing events (Fig. 4C).

Flow cytometry analysis was performed to evaluate both qualitative and quantitative aspects of HDR efficiency. GFP-positive cell populations were clearly distinguishable after genome editing, compared to the negative control (Fig. 4D). To further validate the knock-in of GFP into the viral genome, we performed genomic PCR and Western blot analysis. Primers flanking the knock-in site (F & R) and internal GFP primers (G & R) generated the expected amplicons in GFP-positive virus-infected cells, but not in wild-type HCMV (Toledo) (Fig. 4E). Furthermore, Western blot analysis using GFP-specific antibodies confirmed the expression of GFP at the expected molecular weight, supporting successful insertion and expression of the GFP sequence (Fig. 4F).

Together, these data demonstrate that optimizing the donor-to-Cas9 IDLV ratio and increasing the MOI of HCMV significantly boost HDR outcomes. The synergistic effect of increased donor template availability and higher viral replication likely contributed to the enhanced precision and efficiency of genome editing observed in this system.

### Initial validation of genome editing by GFP fusion to IE2 protein

**Fig. 5.**
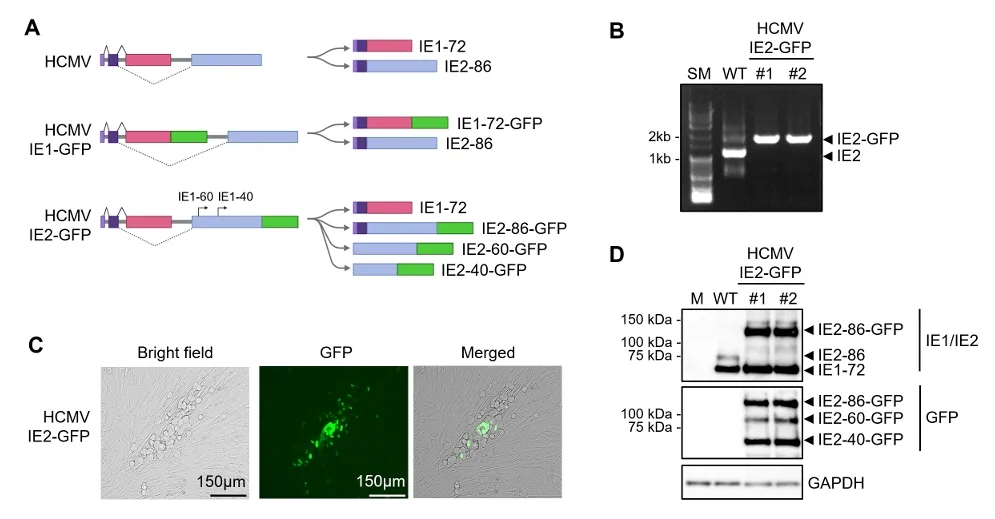
Generation and validation of HCMV IE2-GFP recombinant virus. (A) Schematic representation of the genomic region encoding the IE1 and IE2 proteins in HCMV, highlighting their alternative splicing patterns. The GFP fusion site is indicated by green boxes at the C-terminus of each protein isoform. (B) PCR genotyping confirms successful GFP integration at the IE2 locus in genome-edited HCMV. Results are shown for the wild-type (WT) and two independent recombinant clones (#1 and #2). (C) Fluorescence microscopy images demonstrating GFP expression in cells infected with the recombinant HCMV IE2-GFP virus. (D) Western blot analysis validating the expression and correct fusion of GFP to the C-terminus of IE2 isoforms. Antibodies specific for IE1/IE2 and GFP detected the expected fusion proteins. GAPDH was used as a loading control.

To initially validate our CRISPR-Cas9 HDR system, we generated recombinant viruses expressing GFP fused directly to the IE1 protein (Fig. 4E and 4F), which is non-essential for HCMV replication. Following successful validation of this initial approach, we proceeded to target the essential IE2 gene, generated from the same genomic region by alternative splicing (Fig. 5A). Recombinant viruses expressing IE2-GFP were successfully isolated after six rounds of plaque purification, with genomic integration confirmed by PCR and sequencing, and protein expression verified by Western blot (Fig. 5B–5D). Western blot analysis revealed that GFP was fused to all major IE2 isoforms (IE2-86, IE2-60, and IE2-40), indicating that C-terminal tagging was preserved across alternatively spliced transcripts (Fig. 5D).

Taken together, these results demonstrate the high accuracy and efficiency of our genome editing system, highlighting its ability to precisely fuse GFP to the essential IE2 protein without disrupting alternative splicing or isoform diversity.

### Validation of the IE1-AID-P2A-GFP knock-in and auxin-induced degradation

**Fig. 6.**
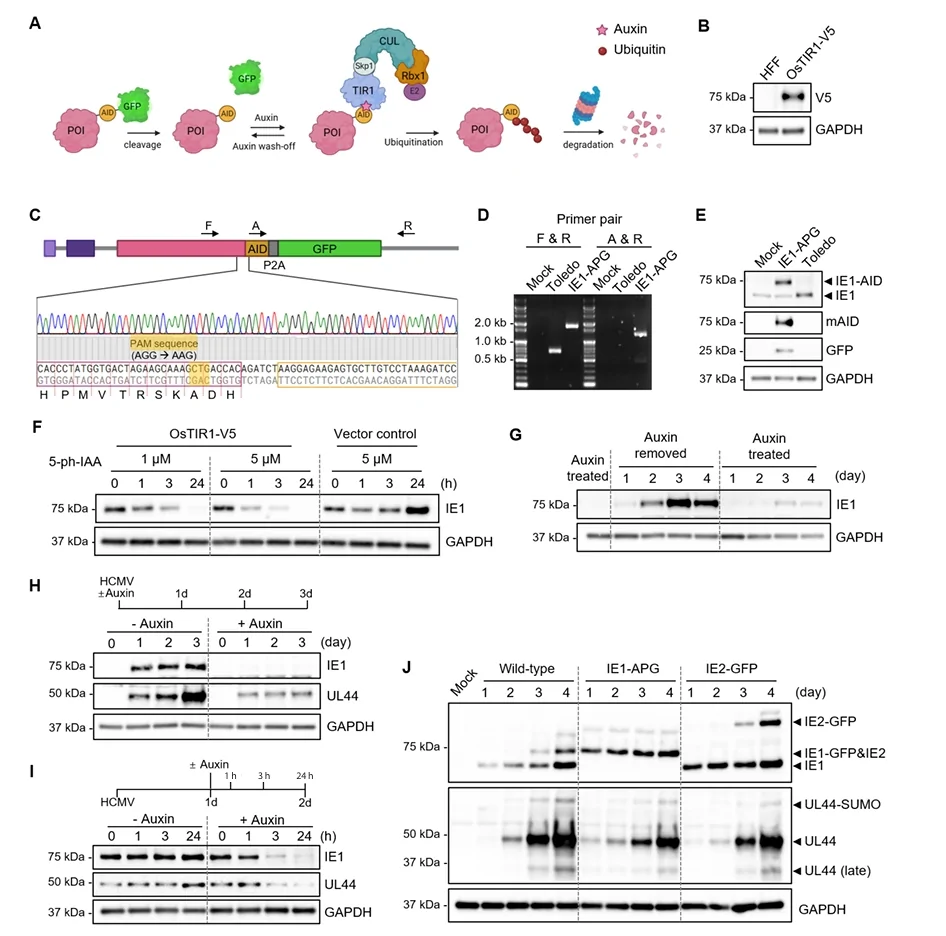
Generation and validation of recombinant HCMV IE1-APG virus. (A) Schematic diagram illustrating the AID-P2A-GFP (APG) system. Upon translation, the P2A peptide mediates ribosomal skipping, resulting in the separation of GFP from the IE1-AID fusion protein. In the presence of auxin, the AID-tagged IE1 interacts with OsTIR1, leading to polyubiquitination and proteasomal degradation of the target protein. (B) Western blot confirming the expression of OsTIR1-V5 in engineered host cells. (C) Schematic of the IE1-APG knock-in strategy and genomic structure. Arrows indicate the positions of PCR primers (F & R and A & R) flanking the insertion site. (D) PCR analysis confirming successful integration of the APG cassette into the IE1 locus. Amplicons generated using F & R primers were cloned and sequenced. Sequencing results confirmed accurate in-frame insertion and PAM site substitution (AGG → AAG), preserving the lysine codon while preventing Cas9 re-cleavage. (E) Western blot validating proper expression and processing of the IE1-APG construct. Antibodies against IE1, AID, and GFP detected both the full-length IE1-AID fusion protein and cleaved GFP, confirming appropriate expression and post-translational cleavage. GAPDH was used as a loading control. (F) Western blot showing rapid degradation of IE1-AID upon treatment with different concentrations (1 µM or 5 µM) of 5-ph-IAA, an auxin analog. HCMV IE1-APG-infected cells expressing or lacking OsTIR1-V5 were treated with auxin for 0, 1, 3, and 24 h. (G) Time-course analysis of the reversibility of AID-mediated degradation. After 24 h of auxin treatment, the compound was removed, and cells were incubated for an additional 1 to 4 days. Western blot analysis shows progressive restoration of IE1-AID levels, confirming the reversible nature of the APG system. (H–I) Western blot analysis showing time-dependent degradation of IE1-AID and the corresponding reduction in UL44 expression following auxin treatment. Cells expressing OsTIR1-V5 were infected with HCMV IE1-APG and treated with 5-ph-IAA (5 µM) at the time of infection (H) or 1 day post-infection (I). Cells were harvested at the indicated time points after treatment. DMSO (0.1%) was used as the vehicle control (– auxin). (J) Western blot analysis comparing the expression kinetics of viral proteins among wild-type, IE1-APG, and IE2-GFP HCMV strains. HFF cells were infected with each virus at an MOI of 1, and whole-cell lysates were collected at 1, 2, 3, and 4 days post-infection.

To enable precise temporal control of IE1 protein levels, we inserted an auxin-inducible degron (AID) system at the C-terminus of IE1. The APG (AID-P2A-GFP) cassette was designed to allow for both visual tracking (via GFP) and auxin-dependent degradation (via AID), separated by a self-cleaving P2A peptide (Fig. 6A). In the presence of auxin and the auxin receptor TIR1, the AID-tagged protein is recognized by the SCF ubiquitin ligase complex, is ubiquitinated and subsequently degraded by the proteasome complex (Fig. 6A and 6B). To prevent repeated cleavage by Cas9 following successful HDR, we introduced a silent mutation in the PAM sequence (AGG to AAG) without altering the amino acid (Lys) sequence, as verified by Sanger sequencing (Fig. 6C). Genomic PCR using primers flanking the IE1 C-terminus confirmed successful integration of the APG cassette, as shown by the appearance of the expected amplicon size only in the knock-in virus (Fig. 6D). Western blot analysis using IE1- and AID-specific antibodies confirmed the expression of the IE1-AID fusion protein in infected cells. In line with the P2A design, free GFP was also detected as a separate band, validating successful post-translational cleavage (Fig. 6E).

To functionally assess the AID system, HFF cells stably expressing the TIR1-V5 receptor (Fig. 6B) were infected with the HCMV IE1-APG virus. Upon treatment with 5-ph-IAA, IE1-AID protein levels decreased rapidly, with degradation detectable as early as 1 h post-treatment and near-complete depletion observed by 24 h (Fig. 6F). Following auxin removal, IE1-AID protein levels gradually recovered over the course of four days, demonstrating the reversible nature of this system (Fig. 6G). To investigate the phenotypic impact of temporal IE1 depletion on viral replication, we treated IE1-APG-infected cells with auxin either at the time of infection (Fig. 6H) or at one day post-infection (Fig. 6I), and monitored the expression of UL44, a DNA polymerase processivity subunit essential for HCMV replication. Interestingly, auxin treatment at the time of infection prevented UL44 accumulation throughout the course of infection, indicating that early expression of IE1 is required to initiate replication-associated gene expression. In contrast, a gradual decrease in UL44 expression was observed following IE1 depletion. These results suggest that IE1 not only functions during the immediate-early phase but also plays a sustained regulatory role in supporting viral gene expression and replication during later stages. These findings further highlight the utility of our inducible degradation system for dissecting the temporal functions of viral proteins.

To evaluate the potential impact of fusion tags on viral protein function, we compared the expression kinetics of IE1, IE2, and UL44 in cells infected with wild-type, IE1-AID, or IE2-GFP viruses (Fig. 6J). The timing and levels of protein accumulation were comparable across strains, suggesting that, at least for IE1 and IE2, the fusion tags did not markedly impair function or replication.

Together, these findings demonstrate the utility of the auxin-inducible degron system as a versatile tool for temporally regulating critical viral regulators, enabling functional dissection of gene networks involved in replication and pathogenesis with minimal disruption to viral fitness.

## Discussion

Our CRISPR-Cas9-mediated gene editing system represents a significant advancement in HCMV genome engineering, enabling precise genetic modifications without the need for BAC-based recombination. A key innovation of this study is the strategic use of integrase-deficient lentiviruses (IDLVs) and optimized HDR donor templates containing CRISPR cut sites, which together significantly enhanced recombination efficiency. These improvements make CRISPR-based genome editing more efficient, scalable, and practical for functional virology studies. In addition, our optimization experiments showed that the timing of HCMV infection relative to IDLV delivery also influenced editing efficiency. Simultaneous co-infection produced the most consistent and efficient editing outcomes, while sequential infection modestly reduced efficiency and significantly prolonged the overall workflow.

Notably, we achieved HDR efficiencies far exceeding those typically observed in mammalian genome editing, which generally remain below 10% (Leal et al., 2024). This striking improvement likely results from both the unique replication biology of HCMV and our refined editing strategy. HCMV replication compartments are spatially segregated from host chromatin and may facilitate the localized activity of CRISPR-Cas9 and HDR machinery. Moreover, donor templates flanked by CRISPR cut sites likely enhance the proximity and accessibility of the repair sequence, promoting precise genome integration. The use of IDLVs further extended the availability of the donor template and favored HDR over non-homologous end joining (NHEJ), thereby improving editing precision.

Using this optimized system, we successfully inserted the APG (AID-P2A-GFP) cassette into the HCMV genome, allowing real-time visualization of protein expression while minimizing structural disruption to the native protein through P2A-mediated self-cleavage. This strategy enables precise temporal regulation of protein degradation, particularly valuable for analyzing viral genes that cannot be deleted using conventional knockout methods due to their essential roles in the viral life cycle. Our results confirmed that the AID system provides rapid, reversible, and specific control of protein stability during infection.

To evaluate the functional consequences of temporally controlled degradation, we treated infected cells with auxin at different stages and monitored UL44, a key replication-associated protein. Auxin treatment at the time of infection resulted in sustained depletion of IE1 and complete failure of UL44 accumulation, indicating that early IE1 expression is critical for the initiation of viral replication. In contrast, auxin treatment at one day post-infection led to a gradual decline in UL44 expression, suggesting that IE1 also contributes to maintaining replication during later stages. These findings demonstrate that our inducible system enables temporal dissection of viral protein functions across multiple phases of infection.

To ensure that the insertion of fusion tags did not compromise viral protein function, we compared the expression kinetics of viral proteins between wild-type and recombinant viruses encoding IE1-AID or IE2-GFP. The recombinant strains exhibited comparable temporal patterns of replication protein expression, such as UL44, indicating that the fusion constructs retained sufficient native activity to support viral replication (Fig. 6J).

Taken together, our findings establish a robust genome editing pipeline for HCMV that enables scarless, high-efficiency knock-in modifications. Importantly, this approach is compatible with clinical isolates and does not rely on BAC technology, making it broadly accessible and translationally relevant. By incorporating inducible degron systems such as APG, this platform facilitates the temporal and functional dissection of viral proteins involved in replication, latency, and pathogenesis. This methodology may also be adaptable to other large DNA viruses and inform future genome engineering strategies.

## Figures and Tables

**Table 1.** List of primers used in this study

Name	Sequence (5'-3')
IE1 sgRNA F	CACCGCCCTATGGTGACTAGAAGCA
IE1 sgRNA R	AAACTGCTTCTAGTCACCATAGGGC
IE1 HAS1 F for IE1-GFP	GAAGCAAAGCTGACCACAGATCTATGGTGAGCAAGGGCGAG
IE1 HAS1 R for IE1-GFP	CATAACAGTAACTGATATATATACAATAGTTTACTTGTACAGCTCGTCCATGC
IE1 HAS2 F for IE1-GFP	AGATCTGTGGTCAGCTTTGCTTC
IE1 HAS2 R for IE1-GFP	TAAACTATTGTATATATATCAGTTACTGTTATGGATCC
IE1 HAS1 F for IE1-APG	CCTTGCTTCTAGTCACCATAGGGGCAGAACATGTATGAGAACTACATTG
IE1 HAS1 R for IE1-APG	GGTGGTGGGTTCCTCAGCACC
IE1 HAS2 F for IE1-APG	TAAATAAACTATTGTATATATATCAG
IE1 HAS2 R for IE1-APG	CCTTGCTTCTAGTCACCATAGGGTTCATGATATTGCGCACCTTCTC
pLenti vector R w/ IE1 cut site	CCCTATGGTGACTAGAAGCAAGGGGAACTCCCAAGCTTATCG
pLenti vector F w/ IE1 cut site	CCCTATGGTGACTAGAAGCAAGGTACCGGTTAGTAATGATCGACAATC
IE1 PAM sequence mutagenesis F	CCTATGGTGACTAGAAGCAAAGCTGACCACAGATCTAA
IE1 PAM sequence mutagenesis R	TTAGATCTGTGGTCAGCTTTGCTTCTAGTCACCATAGG
D64V integrase mutagenesis F	ATATGGCAGCTAGTTTGTACACATTTAGAAGGA
D64V integrase mutagenesis R	TCCTTCTAAATGTGTACAAACTAGCTGCCATAT
IE2 sgRNA F	CACCGAGTCTCAGTAAGTGAAAAAC
IE2 sgRNA R	AAACGTTTTTCACTTACTGAGACTC
pLenti vector F w/ IE2 cut site	AGTCTCAGTAAGTGAAAAACTGGTACCGGTTAGTAATGATCGACAATC
pLenti vector R w/ IE2 cut site	AGTCTCAGTAAGTGAAAAACTGGGGAACTCCCAAGCTTATCG
IE2 HAS1 F for IE2-GFP	CCAGTTTTTCACTTACTGAGACTACACCCGCAATCATGAGG
IE2 HAS1 R for IE2-GFP	AGATCTCTGAGACTTGTTCCTCAG
IE2 HAS2 F for IE2-GFP	TAAGTGAAAAACTGGAAAGAGAGACATGG
IE2 HAS2 R for IE2-GFP	CCAGTTTTTCACTTACTGAGACTAGACGGCGTATAGGAGTCC
GFP F for IE2 and IE2-GFP	GAGGAACAAGTCTCAGAGATCTATGGTGAGCAAGGGCGAG
GFP R for IE2 and IE2-GFP	CTCTTTCCAGTTTTTCACTTACTTGTACAGCTCGTCCATGC
WPRE qPCR F	GGCTGTTGGGCACTGACAAT
WPRE qPCR R	CCGAAGGGACGTAGCAGAAG
TIR1(F74G) F	GGGGACAAGTTTGTACAAAAAAGCAGGCTTCATGACATACTTTCCTGAAGAGG
TIR1(F74G) R	GGGGACCACTTTGTACAAGAAAGCTGGGTACAGAATCTTCACAAAGTTGGGA
IE1 genotyping F	CATGAAGGTCTTTGCCCAGT
IE1 genotyping R	CTGCTAACGCTGCAAGAGTG
mAID2 F	AGATCTAAGGAGAAGAGTGCTTGTCC
